# A Canine Model of Hemorrhagic Transformation Using Recombinant Tissue Plasminogen Activator Administration After Acute Ischemic Stroke

**DOI:** 10.3389/fneur.2019.00673

**Published:** 2019-06-25

**Authors:** Run-Hao Jiang, Qing-Quan Zu, Xiao-Quan Xu, Bin Wang, Ye Ding, Jun Wang, Sheng Liu, Hai-Bin Shi

**Affiliations:** ^1^Department of Radiology, The First Affiliated Hospital of Nanjing Medical University, Nanjing, China; ^2^Charles T. Dotter Department of Interventional Radiology, Oregon Health and Science University, Portland, OR, United States; ^3^Department of Maternal, Child and Adolescent Health, School of Public Health, Nanjing Medical University, Nanjing, China; ^4^Department of Toxicology, School of Public Health, Nanjing Medical University, Nanjing, China

**Keywords:** blood-brain barrier, canines, hemorrhagic transformation, ischemic stroke, MMP-9

## Abstract

Early reperfusion of occluded arteries via recombinant tissue plasminogen activator (rtPA) administration is considered to be an effective strategy for the treatment of acute ischemic stroke. However, delayed administration of rtPA may cause severe hemorrhagic transformation (HT) and undesirable neurological outcomes. The current study aims to establish a canine HT model using rtPA administration and to investigate the potential mechanisms underlying HT. Following anesthesia, two autologous clots were injected into the middle cerebral artery (MCA) to induce ischemic stroke. To induce reperfusion, rtPA (2 mg/kg) was administrated intravenously 4.5 h after the establishment of stroke. The occurrence of HT was determined by computed tomography (CT) and by pathological assessment. Transmission electron microscopy was utilized to assess blood-brain barrier (BBB) damage. The expression of matrix metalloprotein 9 (MMP-9) was analyzed by enzyme linked immunosorbent assay (ELISA), immunofluorescence (IF), and western blot. Administration of rtPA 4.5 h after stroke induced reperfusion in 73.9% of the canines, caused evident HT, and did not improve neurological outcomes compared to canines that did not receive rtPA. There was a significant increase in expression of MMP-9 after rtPA administration, accompanied by BBB disruption. We have established a canine HT model that closely mimics human HT by using rtPA administration after the induction of middle cerebral artery occlusion (MCAO) with autologous clots. Our data suggest that a potential mechanism underlying rtPA-caused HT may be related to BBB dysfunction induced by an increase in MMP-9 expression.

## Introduction

Acute ischemic stroke is a leading cause of death and disability worldwide (1). Early administration of the thrombolytic agent recombinant tissue plasminogen activator (rtPA) is the primary strategy for the treatment of ischemic stroke (2, 3). However, in addition to its narrow therapeutic time window (within 4.5 h for intravenous administration after stroke onset), rtPA may induce hemorrhagic transformation (HT). HT is a common and harmful complication of thrombolytic treatment, and can negatively affect neurological outcomes. It has been reported that HT occurs in 20–40% of patients that receive rtPA treatment (4, 5). Due to these limitations, few stroke patients can truly benefit from rtPA-mediated thrombolysis. Thus, there is an urgent need to analyze the molecular mechanisms underlying rtPA-induced HT in order to improve the safety of rtPA administration and advance its clinical application.

Experimental models of cerebral infarction have significant limitations. Many groups have utilized an intraluminal monofilament (6), an injection of clots (7), or a photochemical reaction with rose bengal to induce focal ischemia in rodents (8). To induce HT, these approaches require the use of hypertensive or hyperglycemic rodents (9, 10). Furthermore, a rodent mechanical occlusion model, achieved via middle cerebral artery occlusion (MCAO) by monofilament insertion, is not appropriate to evaluate the efficacy of thrombolytic agents. Moreover, when using a photochemical reaction to establish MCAO, the emboli mainly consist of platelets and lack fibrin, which is not accessible to thrombolysis with rtPA. Additionally, though models that utilize injected clots in rodents closely mimic the pathophysiology of human stroke patients, the external carotid artery (ECA) must be permanently ligated after the injection and may cause hemodynamic changes. Lastly, although preclinical studies of HT using hypertensive or hyperglycemic rodents are valuable to mimic specific conditions such as hypertension or diabetes mellitus, they do not adequately recreate the clinical presentation of HT induced by thrombolytic treatments.

We recently described an endovascular canine stroke model based on MCAO with autologous clots that is highly suitable for the study of ischemic stroke (11). Using this method as a starting point, the current study aims to establish a reproducible and feasible canine model of HT using rtPA administration, and to investigate the potential mechanisms linking HT and rtPA.

## Materials and Methods

### Ethics

All animal-related experiments were performed according to the National Institutes of Health guide for the care and use of laboratory animals. The experimental protocols were approved by the Committee on the Ethics of Animal Experiments, Southeast University Medical School. All experiments, documentation and reporting were in compliance with the ARRIVE guidelines (Animal Research: Reporting *in vivo* Experiments).

### Animals

**Figure 1:**
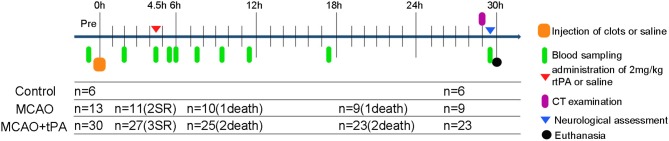
Schematic drawing of experimental protocols. Blood samples were obtained at multiple time points: prior to MCAO; 2, 4.5 h post-stroke; 0, 0.5, 2, 4, 6, 12, and 24 h after rtPA administration. SR, spontaneous reperfusion.

Forty-nine male beagle dogs (10–15 kg, 2–3 years) were acclimatized to our animal facilities for 1 day before the initiation of experiments. Using a table of random numbers, canines were randomly divided into 3 groups: control (sham operation), MCAO, and MCAO + rtPA (Figure 1). Animals from the experimental group were treated and assed first, followed by control group.

Canines were anesthetized with pentobarbital (30 mg/kg) (Chemical Reagent Company, Shanghai, China) and maintained (dose = 1/5 of induction) via administration once every 2 h. Fentanyl (0.03 mg/kg) was used for analgesia peri-operation and post-operation. Physiological parameters, including mean arterial blood pressure (MABP) and blood gas were measured before and after rtPA administration (Supplementary Table 1). Endovascular canine MCAO was performed as previously described (12). Briefly, common femoral artery and vein accesses were achieved using 5-French sheaths (Terumo Medical Corporation, Tokyo, Japan). A bolus of 2,500 U of heparin was given and an intravenous saline infusion (2-mL/min) was maintained through femoral vein access.

**Figure 2:**
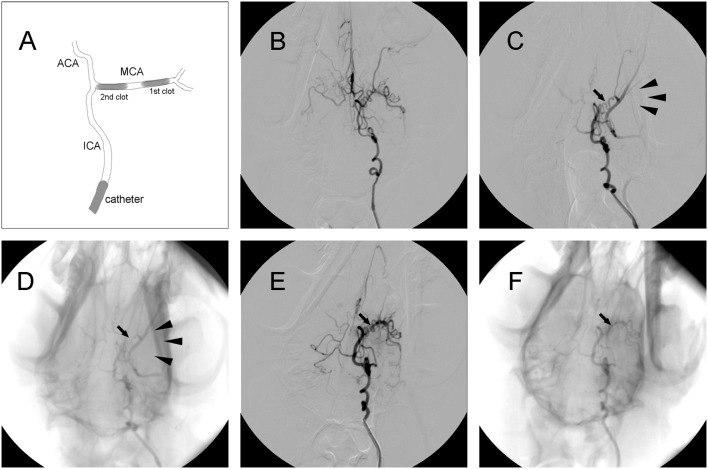
Representative digital subtraction angiography (DSA) images of intracranial arteries in canines. **(A)** Diagram of middle cerebral artery occlusion (MCAO). **(B)** Representative cerebrovascular anatomy. **(C,D)** MCA trunk occlusion (arrow) after clot injection. There was no collateral flow (arrowhead) to the MCA territory. **(E,F)** Recanalization of the MCA (arrow) after administration of rtPA.

Thread-like clots were prepared as previously described (11). Plasma was mixed with thrombin in a customized glass tube and incubated at 37°C for 2 h. Subsequently, clots were cut into segments ~1.4 or 1.7 mm in diameter and 5 mm in length. A 5-French vertebral catheter was inserted into cerebral arteries under fluoroscopic guidance (Axiom Artis, Siemens, Munchen, Germany). After baseline arteriography was performed, the catheter was inserted into the internal carotid artery (ICA). Then, a 1.4 mm diameter clot was placed into a 2-mL syringe filled with contrast agent (Omnipaque 300; GE Healthcare, USA). After the clot was injected into the ICA, the 2-mL syringe was replaced with a 5-mL syringe filled with saline, which was injected into the ICA slowly with intermittent pressure. If the distal M1 segment of the middle cerebral artery (MCA) was occluded, then a 1.7 mm diameter clot was injected to occlude the proximal region of the M1 segment. Angiography was performed to confirm the occlusion of the M1 segment and to evaluate leptomeningeal collateral recruitment. If MCAO was complete, the ipsilateral ICA was blocked using the same catheter, which was connected to pressurized saline for 2 h (Figure 2A).

### rtPA-Induced Thrombolysis

RtPA (Actilyse, Boehringer Ingelheim, Germany) was intravenously administrated at 2 mg/kg with a 10% bolus injection and 90% continuous infusion for 60 min at 4.5 h post-stroke. In canines, the 2 mg/kg dose is considered to equivalent to the clinical dose (in humans) of 0.9 mg/kg after adjusting to the body surface area, as recommended by the Food and Drug Administration guidelines (http://www.fda.gov/Drugs/DrugSafety/ucm223966.htm). The MCAO and control groups received the same volume of saline. The endovascular procedures described above were repeated to investigate whether an effective reperfusion of the MCA was achieved after rtPA administration.

### Computed Tomography (CT)

CT scans were performed using a 16-slice spiral CT scanner (SOMATOM Emotion, Siemens, Germany) according to the following parameters: kV: 120, mAs: 28, and slice thickness: 1.5 mm. CT images were assessed by three blinded investigators.

### Neurological Assessment

All behavioral evaluations were performed by an interventional neuroradiologist who was blinded. A modified canine neurobehavioural scoring system was employed to assess motor function, consciousness, heading turning, circling, and hemianopsia (Supplementary Table 2).

### Hemorrhagic Assessment

Hemorrhagic events were scored as follows: 0 = no blood; 1 = hemorrhagic infarction type 1 (HI-1) (small petechial along the margins of the infarction); 2 = hemorrhagic infarction type 2 (HI-2) (confluent petechial within the infarction); 3 = parenchymal hematoma type 1 (PH-1) (blood clot < 30% of infarction, mild space-occupying effect); 4 = parenchymal hematoma type 2 (PH-2) (blood clot > 30% of infarction, marked space-occupying effect).

### Hematoxylin and Eosin (HE) Staining

Twenty-four hours after thrombolysis, the vertebral catheter was inserted into contralateral ICA, and the dogs were super selectively perfused with saline followed by 4% paraformaldehyde through the catheter. Brains were excised and fixed in 4% paraformaldehyde at 4°C for 24 h, then embedded in paraffin and cut into 50-μm-thick serial sections. Sets of five serial sections were stained with HE.

### Transmission Electron Microscopy (TEM)

Tissue blocks ~1 mm^3^ in size were excised from the peri-infarct region, fixed in 4% glutaraldehyde at 4°C for 24 h, and postfixed in 1% osmium tetroxide for 2 h. Then, the samples were dehydrated through a graded ethanol series, exchanged through propylene oxide, and embedded in epoxy resin at 60°C for 36 h. Ultrathin sections were obtained, stained with 0.5% uranyl acetate and 3% lead citrate, and then observed with a transmission electron microscope (Tecnai G2 Spirit; FEI, Hong Kong, China) at 100 kV.

### ELISA

Blood samples were obtained at the following time points: prior to MCAO; 2, 4.5 h post-stroke; 0, 0.5, 2, 4, 6, 12, and 24 h after rtPA administration (corresponding to 0, 2, 4.5, 5.5, 6, 7.5, 9.5, 11.5, 17.5, and 29.5 h after establishment of stroke). The amount of MMP-9 in serum was quantified using an enzyme linked immunosorbent assay (ELISA) kit (Biofavor, Wuhan, China). Optical density (OD) was detected on a microplate reader at 450 nm and corrected at 540 nm.

### Immunofluorescence (IF)

After fixation in 4% paraformaldehyde at 4°C for 24 h, brains were sequentially dehydrated in 15 and 30% sucrose dissolved in saline. The brains were then embedded in optimum cutting temperature compound (OTC) and sliced into 6-μm-thick coronal sections using a cryostat (Thermo, Boston, MA, USA). Five sections from each animal were stained with anti-MMP-9 (1:100; ab38898, Abcam, Cambridge, UK), followed by staining with secondary antibodies (1:100; BA1032, Cy3 Conjugate Goat Anti-Rabbit IgG, Boster, Wuhan, China). Fluorescence intensities of the different groups were detected by fluorescence microscope (BX53, Olympus, USA).

### Western Blot

Western blotting analysis was performed as described previously (6). The following primary antibodies were used: anti-MMP-9 (1:1000; ab38898, Abcam, Cambridge, UK) and anti-tubulin (1:1000; A5032, Selleck, Houston, TX, USA).

### Statistical Analysis

Data are presented as mean ± standard deviation (SD). To analyse differences between multiple groups, one-way analysis of variance (ANOVA) followed by *post-hoc* least significant difference (LSD) tests was performed (SPSS software; version 22; IBM-SPSS, Inc., Chicago, IL, USA). Results were considered to be statistically significant for *P* < 0.05.

## Results

### General Observations

Spontaneous reperfusion occurred in two beagles of the MCAO group and three of the MCAO + rtPA group before drug administration. The efficacy of embolization in the MCAO and the MCAO + rtPA groups was 81.8 and 90.0%, respectively. There were no significant difference in the mortality rate between the MCAO (2/11) and MCAO + rtPA groups (4/27). A total of 11 canines (five exhibited spontaneous reperfusion, six died before CT examination) were excluded from the experiments (Figure 1). No evidence of severe side effects including gastrointestinal bleeding, pulmonary hemorrhage, and genitourinary hemorrhage were observed.

### Angiographic Findings

Digital subtraction angiography (DSA) clearly defined the anatomy of the cerebrovasculature (Figure 2B). After the injection of two autologous clots, the left MCA trunk was immediately occluded and its territories were no longer visible during DSA analysis (Figures 2C,D). Effective recanalization of the MCA (Figures 2E,F) was achieved in 17 individuals out of 23 (73.9%) in the MCAO + rtPA group after intravenous administration of rtPA.

### Computed Tomography (CT) Scanning

**Figure 3:**
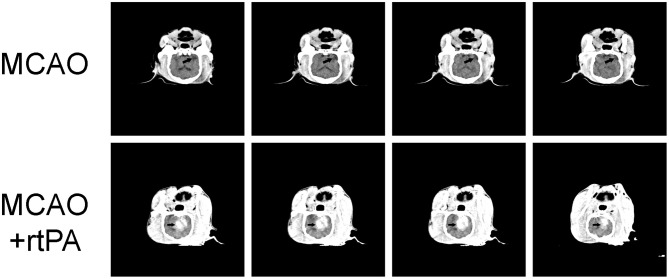
Representative Computed Tomography (CT) images. A low-density infarct area (arrow) in the ipsilateral hemisphere after MCAO. In the MCAO + rtPA group, a high-density region (arrow) was observed.

CT scanning was performed 24 h after rtPA administration. Low-density areas in the cerebral cortex, a typical sign of ischemic cerebral infarction, were observed in the MCAO group. There were obvious high-density regions in the MCAO + rtPA canines (9/23), which indicated the occurrence of significant HT (Figure 3).

### Pathological Findings

**Figure 4:**
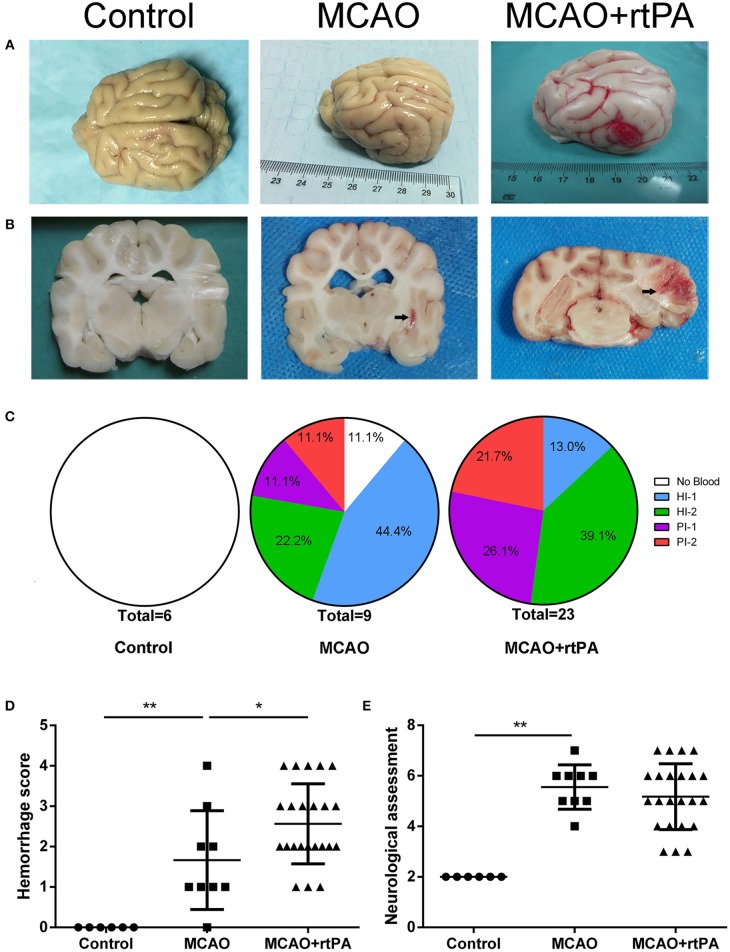
Effect of rtPA administration on macroscopic HT and neurological outcomes. **(A)** General appearance and **(B)** coronal sections of brains from representative experiments. **(C)** Hemorrhages were classified by type and extent in 5 groups: (1) no hemorrhage; (2) HI-1; (3) HI-2; (4) PH-1; (5) PH-2. **(D)** Hemorrhage score and **(E)** neurological assessment were determined in the control, MCAO, and MCAO + rtPA groups (*n* = 6 in the control group, *n* = 9 in the MCAO group, and *n* = 23 in the MCAO + rtPA group). *P < 0.05, **P < 0.01.

**Figure 5:**
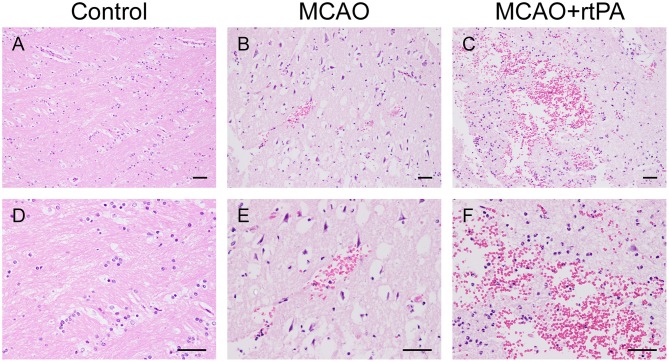
Effect of rtPA administration on microscopic HT. Representative hematoxylin-eosin (HE) staining of brain sections from the control **(A,D)**, MCAO **(B,E)**, and MCAO + rtPA groups **(C,F)**. Red blood cells appear as orange-yellow spherical cells without nuclei. Scale bars = 25 μm.

CT scanning is the primary strategy for the detection of significant HT in the clinic; however, it is less sensitive for the detection of benign HT, such as HI-1 and HI-2. To confirm the presence of HT, pathological analysis was used. In the control group, there was no evidence of HT assessed by the gross pathology (Figures 4A,B) or by histological examination with HE staining (Figures 5A,D). Gross pathological examination revealed that mild signs of HT were present in most canines in the MCAO group (Figures 4A,B). HI-1 was present in 11.1%, HI-2 in 22.2%, PI-1 in 11.1%, and PI-2 in 11.1% of the canines (Figure 4C). Mild bleeding was detected upon HE staining, as well as neuronal pyknosis, degeneration of cell bodies, and infiltration of inflammatory cells (Figures 5B,E).

Gross pathological assessment confirmed the occurrence of significant HT after administration of rtPA 4.5 h post-stroke (Figures 4A,B). Furthermore, rtPA administration increased the incidence of HI-2, PI-1, and PI-2 while reducing that of HI-1 (Figure 4C). In addition, the hemorrhage scores of the MCAO + rtPA group were significantly increased compared to those of the MCAO group (Figure 4D). HE staining revealed pyknotic nuclei and massive blood extravasation into the parenchyma (Figures 5C,F).

### Neurological Outcomes

After recovering from anesthesia, the canines were subjected to neurological assessment using an 11-point neurobehavioral scoring system. There was a significant difference in the neurological scores between the control group and the MCAO group. All the canines in the MCAO group showed neurobehavioral deficits including circling, reduced consciousness, inability to stand, and ipsilateral hemiparesis. rtPA administration at 4.5 h post-stroke did not improve the neurological outcomes observed in the MCAO group (Figure 4E).

### rtPA Administration Disrupted the BBB

**Figure 6:**
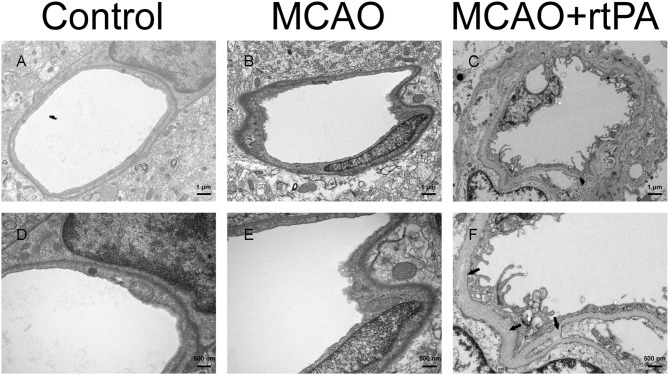
Ultrastructure of the blood–brain barrier. The basement membrane (arrow) and endothelial cells were disrupted in the MCAO group **(B,E)** as compared to the control group **(A,D)**, and the damage was exacerbated after rtPA administration **(C,F)** (*n* = 3–4 per group).

BBB disruption is a central feature of acute ischemic stroke (13), and may also play an important role in HT. Using electron microscopy, we assessed the ultrastructure of the BBB. In the control group, we observed that the basement membrane and tight junctions were intact (Figures 6A,D). After MCAO, the basement membrane was partially damaged while the tight junctions were still visible, the adjacent neuropil displayed cellular edema and cellular debris (Figures 6B,E). After rtPA administration, in addition to the observation of a disintegration of the endothelial layer, we observed the formation of vacuoles, as well as a dramatic increase in the microvilli of endothelial cells (Figures 6C,F).

### rtPA Administration Promoted MMP-9 Expression

**Figure 7:**
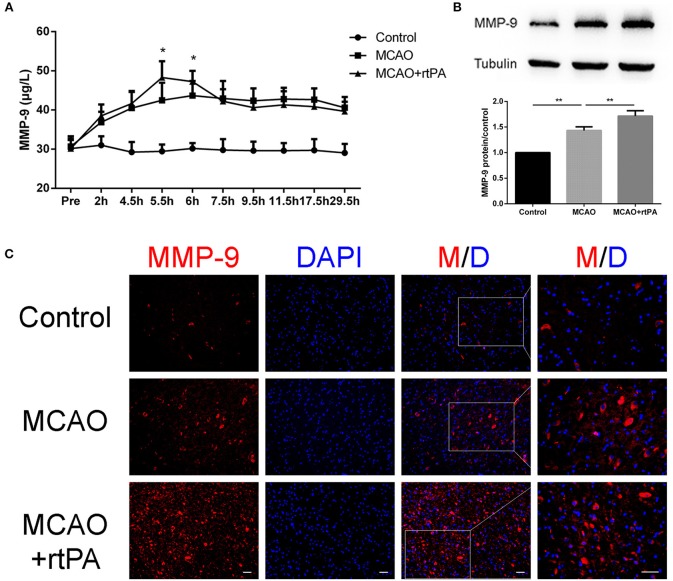
Expression of MMP-9 in the serum and peri-infarct regions. **(A)** Serum MMP-9 levels after thrombolysis (*n* = 6 in the control group, *n* = 9 in the MCAO and MCAO + rtPA group). **(B)** MMP-9 protein expression after MCAO measured by western blot and **(C)** immunofluorescence (*n* = 3–4 per group). Scale bars = 25 μm. *P < 0.05, **P < 0.01.

Matrix metalloprotein 9 (MMP-9) is a well-recognized peptidase that digests components of the basal lamina, and may contribute to BBB damage (14). We analyzed MMP-9 expression in serum at different time points using ELISA. A rapid and significant increase in serum MMP-9 expression was observed in the MCAO + rtPA group compared to the MCAO group. Notably, MMP-9 expression in the MCAO + rtPA group returned to a level similar to that of the MCAO group approximately 2 h after thrombolysis (Figure 7A).

Western blot analysis was utilized to assess MMP-9 protein expression in the peri-infarct region 24 h post-thrombolysis. The results suggested that MCAO significantly increased the MMP-9 expression in this region compared to the control group. This effect was significantly enhanced in the MCAO + rtPA group (Figure 7B).

We further analyzed MMP-9 protein expression in the peri-infarct regions by IF. MMP-9 expression was significantly higher in the MCAO group compared to the control group. In addition, MMP-9 expression was further increased after rtPA administration (Figure 7C).

## Discussion

To date, rtPA is the only FDA-approved drug to treat acute ischemic stroke. Unfortunately, it may cause severe complications such HT (3, 15). The primary purpose of the present study was to induce HT caused by rtPA administration in an endovascular canine stroke model and to elucidate candidate mechanisms underlying HT. Our results unequivocally demonstrate that rtPA administration at 4.5 h post-stroke increases the occurrence of significant HT without improving neurological outcomes. Additionally, after rtPA administration we observed BBB damage and detected enhanced MMP-9 expression by ELISA, IF, and western blot.

In order to better understand HT, an appropriate animal model that reflects the pathogenesis of human HT is urgently needed. Garcia-Yebenes et al. previously described a mouse HT model based on *in situ* clot formation, which was thought to be suitable for investigation of HT caused by rtPA (16). However, MCA puncture, which is indispensable for this methodology, may lead to cerebrospinal fluid leakage (CSFL). Some biochemical parameters might be affected by CSFL, especially those implicated in edema. Additionally, CSFL can induce meningitis and encephalitis, which may extremely affect the results. In a photothrombotic model, vascular endothelium injury caused by photochemical reaction may lead to vascular leakage (17), and the emboli are platelet-rich and lack fibrin. Hence, this model is suitable for studies on antiplatelet drugs and endothelial cell protective agents, but it is problematic when using rtPA.

In the current study, we induced MCAO in canines with an injection of two autologous clots followed by intravenous administration of rtPA, which closely resembles the features of a typical clinical situation. Interestingly, nearly half of the canines in the MCAO group presented with very mild HT (HI-1), as detected by pathological examination, while the rest of the MCAO group exhibited mild bleeding (classified as HI-2, PI-1). These results suggest that MCAO itself can lead to HT even in the absence of rtPA administration, which is consistent with the findings of Couret et al. (10). Our results suggest that rtPA administration at 4.5 h post stroke remained effective for the induction of MCA recanalization, but neurobehavioral scores were not improved. Moreover, higher HT incidence was observed along with a significant increase in hemorrhage scores. Since the blood pressure and blood sugar levels were generally within the normal range, the above mentioned HT was most likely caused by rtPA thrombolysis, rather than hypertension or hyperglycemia. However, this is still a safe time when it translates to humans, likely due to species differences.

The current model presents various advantages. First, as a large animal with a gyrencephalic brain, the vessel size, and degree of vasospasm encountered in canines are more similar to humans compared to rodents, and it is more convenient to conduct imaging studies on canines because of their compatibility with non-specialized, clinical imaging equipment (18–20). Second, unlike laser Doppler flowmetry, fluoroscopy can guide the injection of clots in real time and provide direct evidence of effective thrombolysis. Third, the endovascular procedures that we used represent a minimally invasive technique that avoids unnecessary damage such as craniotomy, muscle incision, and dissection of ECA. The stress and inflammation caused by these damage may affect the results of the study. Finally, the fibrin-rich emboli used in our model resemble those present during cardiogenic cerebral infarction in humans and are appropriate for the study of rtPA-mediated thrombolysis. Using our model as a starting point, future studies can adjust the components of the emboli to mimic different clinical situations, such as ischemic stroke secondary to atherosclerosis. Since our model is able to closely mimic a range of clinical situations, it is suitable for the study of the adverse effects of rtPA treatment and for the investigation of the mechanisms underlying HT.

HT is defined as the extravasation of blood into brain tissue, and may be associated with BBB breakdown (21, 22). The BBB plays a critical role in the maintenance of the internal environment of the central nervous system. BBB breakdown is thought to be a prerequisite for cerebral edema and HT (23). In agreement with the findings of Cai et al. (24), we observed more significant BBB damage in rtPA-treated canines compared to those in the MCAO group.

MMP-9, a member of the matrix metalloprotein family, is a zinc-dependent enzyme that digests the extracellular matrix. Its substrates include collagen IV, laminin, and fibronectin (25–27). We hypothesized that BBB dysfunction is highly related to MMP-9 expression. To further investigate the mechanisms underlying HT, we evaluated the expression of MMP-9. Consistent with results from other groups (28, 29), we observed that the levels of MMP-9 in serum were immediately increased after rtPA administration, which suggests that rtPA can enhance the expression of MMP-9 in peripheral blood.

Though there were no significant differences in serum MMP-9 levels between the MCAO group and the MCAO + rtPA group at 29.5 h post-stroke, the IF and western blot results confirmed an increase in MMP-9 protein levels in the peri-infarct region in the MCAO + rtPA group at this time point. Many studies have indicated that neutrophils are the main source of MMP-9 in peripheral blood (30–32). Accordingly, we hypothesized that intravenous administration of rtPA may induce the expression of MMP-9 in neutrophils in peripheral blood, which then digest the extracellular matrix and penetrate into the cerebral tissue. Once the neutrophils enter the brain tissue, they in turn cause increased MMP-9 expression in resident brain cells such as neurons, astroglia, and microglia leading to further neurological damage. Clearly, further experiments focused on identifying the cellular source of MMP-9 are required. Our results collectively indicate that rtPA administration significantly increases MMP-9 expression, causes secondary dysfunction to the BBB after MCAO, and induces HT.

There are some limitations of the current study: (1) Due to the high density of bleeding and the unclear infarct boundary in CT scans, we didn't measure infarct volume in our research. To overcome this obstacle, we utilized an 11-point neurological score to assess the therapeutic effects of rtPA administration. In future studies, other methods such as multimodality magnetic resonance imaging may be utilized to resolve this deficiency. (2) To simulate clinical situation, we set rtPA administration at 4.5 h post stroke. For optimization of this model or exploration of time-dependent effects of rtPA, further studies could chose more time points for rtPA thrombolysis. (3) In order to prevent excessive animal deaths, the observation time after rtPA thrombolysis is relatively short. With an increased observation time, the occurrence HT could also increase. (4) There is a lack of specific antibodies in canines, as is the case with many large experimental animals. As a result, though complicated interactions between MMPs and tissue inhibitor of metalloproteinase have been previously described (28, 33, 34), we cannot study these relationships. Advances in biotechnology and antibody production will help to alleviate these problems in the future.

## Conclusion

In summary, we have established a reproducible and feasible canine model of HT caused by rtPA administration. BBB dysfunction caused by rtPA-induced MMP-9 expression is a candidate mechanism for HT induction by rtPA.

## Data Availability

The raw data supporting the conclusions of this manuscript will be made available by the authors, without undue reservation, to any qualified researcher.

## Ethics Statement

All animal-related experiments were performed according to the National Institutes of Health guide for the care and use of laboratory animals. The experimental protocols were approved by the Committee on the Ethics of Animal Experiments, Southeast University Medical School. All experiments, documentation, and reporting were in compliance with the ARRIVE guidelines (Animal Research: Reporting *in vivo* Experiments).

## Author Contributions

R-HJ, Q-QZ, X-QX, SL, and H-BS conceived the project and designed the experiments. R-HJ, Q-QZ, and X-QX performed experiments. BW, YD, and JW contributed to the analysis and interpretation of data. R-HJ wrote the manuscript. All authors revised the work critically and approved the final version of the manuscript.

### Conflict of Interest Statement

The authors declare that the research was conducted in the absence of any commercial or financial relationships that could be construed as a potential conflict of interest.
