# Supplementary material for: A Canine Model of Hemorrhagic Transformation Using Recombinant Tissue Plasminogen Activator Administration After Acute Ischemic Stroke
Source: Front Neurol. 2019 Jun 25;10:673. doi: 10.3389/fneur.2019.00673 (PMC6603151; doi:10.3389/fneur.2019.00673)
Supplement: Supplementary file 1 [file Table_1.DOCX]

**Supplementary table 1.** Physiological parameters.

| Variables | Control | MCAO | |  | MCAO + rtPA | |
| --- | --- | --- | --- | --- | --- | --- |
|  |  | prior to  ischemia | 29.5 h after  ischemia |  | prior to  ischemia | 29.5 h after  ischemia |
| Na (mmol/L) | 139 ± 6.6 | 140.5 ± 7.8 | 142 ± 9.2 |  | 140 ± 5 | 137.8 ± 6.8 |
| Hb (g/dL) | 12.8 ± 2.2 | 14.5 ± 1.7 | 14.6 ± 1.8 |  | 12.7 ± 1.7 | 12.4 ± 1.7 |
| pH | 7.34 ± 0.04 | 7.34 ± 0.03 | 7.24 ± 0.06 |  | 7.33 ± 0.05 | 7.28 ± 0.06 |
| PCO_2_(mmHg) | 40 ± 4.1 | 42.9 ± 8 | 44.5 ± 11.9 |  | 40.9 ± 8.4 | 42.8 ± 7.8 |
| PO_2_ (mmHg) | 98 ± 4.1 | 97.8 ± 8.2 | 88 ± 6.2 |  | 98 ± 9.5 | 90.3 ± 12.2 |
| SO_2_ | 97 ± 1.2 | 96.2 ± 1.5 | 94.8 ± 1.5 |  | 97.3 ± 0.8 | 96.8 ± 1.1 |
| MABP(mmHg) | 102.8 ± 4.8 | 103.3 ± 4.9 | 100.1 ± 5.0 |  | 101.3 ± 5.5 | 98.7 ± 5.7 |
| Glu(mmol/L) | 4.87 ± 0.48 | 5.30 ± 0.50 | 4.97 ± 0.74 |  | 5.10 ± 0.32 | 4.92 ± 0.71 |

Values are mean ± SD (n = 3–5 per group). Hb = hemoglobin; MABP = Mean arterial blood pressure; Glu = Blood glucose.
