# Supplementary material for: A Canine Model of Hemorrhagic Transformation Using Recombinant Tissue Plasminogen Activator Administration After Acute Ischemic Stroke
Source: Front Neurol. 2019 Jun 25;10:673. doi: 10.3389/fneur.2019.00673 (PMC6603151; doi:10.3389/fneur.2019.00673)
Supplement: Supplementary file 2 [file Table_2.DOCX]

**Supplementary table 2.** Modified canine neurobehavioral scoring system

| Score | Motor function | Consciousness | Heading turning | Circling | Hemianopsia |
| --- | --- | --- | --- | --- | --- |
| 0 |  |  | Absent | Absent | Absent |
|  |  |  |  |  |  |
| 1 | No deficit | Normal | posturing and turns toward side of infarct, coma, dead | Present, does not walk, dead | Present, unable to test because of consciousness |
| 2 | Able to walk | Mildly reduced |  |  |  |
| 3 | Stand only with assistance | Severely reduced |  |  |  |
| 4 | Hemiparetic and unable to stand, comatose or dead | Comatose or dead |  |  |  |
